# Cleaning effect of osteoconductive powder abrasive treatment on explanted human implants and biofilm‐coated titanium discs

**DOI:** 10.1002/cre2.100

**Published:** 2018-02-15

**Authors:** Ceylin S. Tastepe, Xingnan Lin, Arie Werner, Marcel Donnet, Daniel Wismeijer, Yuelian Liu

**Affiliations:** ^1^ Department of Oral Implantology and Prosthetic Dentistry, Academic Centre for Dentistry Amsterdam (ACTA) University of Amsterdam and Vrije Universiteit Amsterdam; ^2^ Affiliated Stomatological Hospital of Medical School Nanjing University, Department of Orthodontics Nanjing China; ^3^ Department of Dental Material Sciences, Academic Centre for Dentistry Amsterdam (ACTA) University of Amsterdam and Vrije Universiteit Amsterdam; ^4^ Research Group, Dental E.M.S. Electro Medical Systems S.A. Switzerland

**Keywords:** air powder abrasive, titanium surface, implant cleaning, calcium phosphate, peri‐implantitis, dental implant cleaning

## Abstract

The aim of this study is to test the cleaning effect and surface modification of a new implant surface treatment on explanted dental implants and titanium discs. It is a modified air powder abrasive (APA) treatment applied using osteoconductive powders. Twenty‐eight in vitro Ca‐precipitated organic film‐coated titanium discs and 13 explanted dental implants were treated. In a 2‐step approach, 3 powders were used: hydroxylapatite (HA) and biomimetic calcium phosphate (BioCaP), which are osteoconductive, and erythritol, which is not. APA treatment was applied. (Air pressure: 2.4 bar; water flow for cleaning: 41.5 ml/min, for Coating 1: 2.1 ml/min, and for Coating 2: 15.2 ml/min.) The test groups were as follows: Group 1: HA cleaning + BioCaP Coating 1; Group 2: HA cleaning + BioCaP Coating 2; Group 3: erythritol cleaning + BioCaP Coating 1; Group 4: erythritol cleaning + BioCaP Coating 2; Group 5: HA cleaning; Group 6: erythritol cleaning; and control: no powder. Cleaned areas were calculated by point counting method. Surface changes and chemical content were evaluated using light microscopy, scanning electron microscopy, and energy‐dispersive X‐ray spectroscopy. Cleaning effect between groups was compared by a pairwise Student's t test. The significance level was fixed at p < .05. Cleaning effect on the discs was 100% in all test groups and 5% in the control. Powder particles in varying size and shape were embedded on the surface. All HA‐ or CaP‐treated surfaces showed Ca and P content but no surface damage. Calcified biofilm remnants were removed from the implant surface by the test groups, whereas in control groups, they remained. APA treatment with CaP and HA powders under clinically applicable pressure settings gives positive results in vitro; therefore, they could be promising when used in vivo.

## 1 INTRODUCTION

The treatment of peri‐implantitis is primarily focused on elimination of the infection and restoration of the original peri‐implant condition (Baron, Haas, Dortbudak, & Watzek, 2000). In the current literature, different approaches are described such as surgical and nonsurgical interventions combined with mechanical (e.g., titanium, plastic, or steel curettes, saline rinse, cotton gauze, and air abrasion) and chemical (e.g., chlorhexidine, tetracycline, metronidazole, and citric acid) implant surface treatments. However, there is no reliable evidence that suggests which could be the most effective interventions (Esposito, Grusovin, & Worthington, 2012). According to the Consensus Report of the Sixth European Workshop on Periodontology, the outcome of nonsurgical treatment of peri‐implantitis is unpredictable. On the other hand, the surgical treatment gives better results because it provides direct access to the implant surface for debridement and decontamination and achieves resolution of the inflammatory lesion (Lindhe & Meyle, 2008).

Because the biofilm is the primary etiological factor, its removal from the implant surface is obligatory for reosseointegration. However, according to the studies done by Persson, Araujo, Berglundh, Grondahl, and Lindhe (1999) and Wetzel, Vlassis, Caffesse, Hämmerle, and Lang (1999) even after biofilm removal, the true reosseointegration is very difficult to achieve on previously contaminated implant surfaces. New bone fill following the peri‐implantitis treatment is possible to a certain degree, but at all experimental implant sites, a thin connective tissue capsule was found to separate the implant surface from the newly formed bone (Persson et al., 1999). On the basis of the outcome of the studies, it was concluded that the problem inherent in the reosseointegration appears to be the implant surface rather than the host tissues at the site (Persson, Berglundh, Lindhe, & Sennerby, 2001). To overcome this problem, implant surface treatment methods should not only remove the biofilm and debris but also restore the initial implant surface properties or even improve them. It is reported that the contamination of the implant surface causes a lack of osteoconductivity (Baier & Meyer, 1988; Kubies, Himmlova, Riedel, et al., 2011), and this may be the reason of the insufficient reosseointegration after the treatment. Therefore, a surface treatment that will improve the osteoconductive properties of the surface might be crucial to achieve reosseointegration.

Air powder abrasive (APA) treatment is one of the mechanical implant surface treatment methods. It uses an abrasive powder introduced into a stream of compressed air (Moene, Decaillet, Andersen, & Mombelli, 2010). The APA technology was developed for the supragingival cleaning or polishing of the natural tooth surfaces. With the development of glycine powders, the subgingival use is also proven to be safe and more efficient than mechanical scaling and root planning in removing the subgingival biofilm in moderate‐to‐deep periodontal pockets (Petersilka, Faggion Jr., Stratmann, et al., 2008; Moene et al., 2010; Wennstrom, Dahlen, & Ramberg, 2011; Flemmig et al., 2012). This method is also tested on titanium surface, and a number of studies showed that it successfully removes the biofilm from the titanium surface without damaging it (Dennison, Huerzeler, Quinones, & Caffesse, 1994; Zablotsky, Diedrich, & Meffert, 1992; Mouhyi et al., 1998; Augthun, Tinschert, & Huber, 1998; Parham Jr. et al., 1989; Schwarz, Ferrari, Popovski, Hartig, & Becker, 2009).

According to a review on several in vitro and in vivo APA studies (Tastepe, van Waas, Liu, & Wismeijer, 2012), the in vitro cleaning effect of the method is reported to be high. The method resulted in minor surface changes on titanium discs. Although the specimens treated with the APA method show sufficient levels of cell attachment and cell viability, the cell response is decreased compared to the sterile discs. Considerable reosseointegration between 39% and 46% and improved clinical parameters are reported in an animal study (Schou, Holmstrup, Jorgensen, et al., 2003) following application when used with surgical treatment. The results of the treatment are influenced by the powder type used, by the application time, and whether it is applied surgically or nonsurgically.

Although the APA treatment has shown promising results for implant surface cleaning, it does not yet support reosseointegration. Therefore, the method can be improved by changing the above‐mentioned parameters. In our previous study, we suggested to apply the APA treatment with osteoconductive (hydroxylapatite [HA], calcium phosphate [CaP], and TiO_2_) powders that would not only remove the biofilm but also improve the surface properties in favor to reosseointegration (Tastepe, Liu, Visscher, & Wismeijer, 2013). The results showed that APA with osteoconductive powders was able to clean the surface using high‐pressure settings.

As a follow up of our previous study, this study is based on the following hypothesis: osteoconductive powder abrasive treatment under safe and intraorally applicable pressure settings can achieve efficient cleaning without damaging the titanium and modify and improve the surface with impacted powder particles. We developed a new in vitro model and modified the APA treatment with new powder mixtures by applying safe pressure and water settings. The cleaning effect and surface modification were checked on Ca‐precipitated organic film‐coated titanium specific leaf area Sand Blasted Large Grit Acid Etch (SLA) surface discs, and the effects were observed on extracted human implants. If the results of this in vitro study are positive, this treatment approach may be promising when used in vivo.

## 2 MATERIALS AND METHODS

### 2.1 Specimens

**Figure 1:**
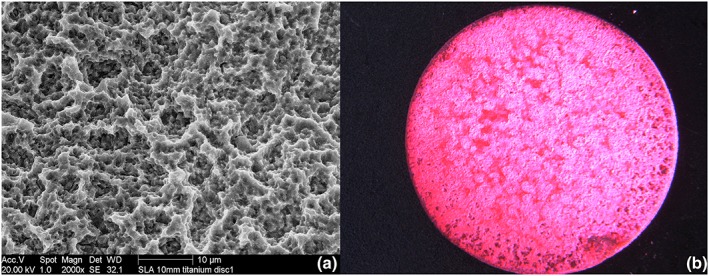
(a) Scanning electron microscopy photo of sandblasted large grit acid etch (SLA) surface of a 10‐mm titanium discs. (b) Light microscope photo of an erythrosine‐stained Ca(OH)₂ accumulated organic film layer

Twenty‐eight titanium discs (titanium CP Grade 3) of 10 mm in diameter (Ningbo Cibei Medical Treatment Appliance Co., Zhejiang, China) were surface treated to create an SLA surface. The discs were sandblasted by 120 μm Al_2_O_3_ particles at 3.9 bar, and acid was etched by using a mixture of 9.5% HCl and 24.5% H_2_SO_4_ at 60 °C for 30 min and then ultrasonically cleaned in distilled H_2_O for 15 min (Figure 1). Following this, the discs were covered with a Ca‐precipitated organic film layer. Sterile titanium discs (SLA surface, Sa: 2.9 μm, contact angle: 111.61°, and 10 mm in diameter) were incubated in α‐minimum essential medium supplemented with 20% fetal bovine serum (FBS) at 37 °C with 5% carbon dioxide and 95% humidity for 24 hr to create a pellicle on the titanium surface and facilitate the biofilm formation. Subsequently, every disc was immersed in 1 ml fresh unstimulated saliva from a healthy donor and incubated for another 24 hr. The next day, fresh α‐minimum essential medium supplemented with 20% FBS was added on each disc and incubated for 72 hr. The medium was refreshed, and the discs were incubated again for 72 hr. The discs were transferred into new well plates containing 2 ml saturated (0.02 M) Ca(OH)_2_ solution and incubated for 72 hr to create Ca precipitation on the discs.

Thirteen commercially available titanium SLA surface implants were collected for the study. All implants were explanted due to peri‐implantitis. The implants were diverse in brand, diameter, and length. The fresh biofilm on one of the implants was fixated by a cell‐drying protocol immediately after the extraction and gold sputtered. High magnification scanning electron microscopy (SEM) photos of this implant were made to visualize the biofilm. The rest of the implants were autoclaved after the extraction without any mechanical cleaning. The surface properties and mineralized remnants were visualized by SEM.

### 2.2 Treatment

All discs were treated by APA treatment. EMS airflow device (AIR‐FLOW master and AIR‐FLOW Perio, EMS, Nyon, Switzerland) was utilized with a standard airflow nozzle. EMS AIR‐FLOW Chamber and Perio Plus Chamber was used for the cleaning and coating step, respectively. The treatment settings are described below. The pressure inside the chamber (DPI 802 P GE Druck), the consumed powder amount, and the water flow were measured during the treatment using a manometer and a balance (Mettler Toledo PR 8002).

**Figure 2:**
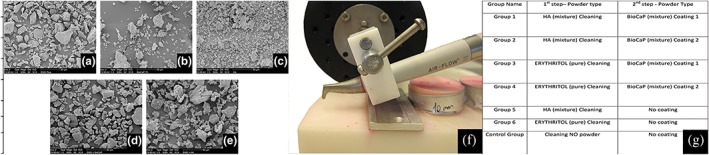
(a through e) Scanning electron microscopy photos of the used powders. (a) Erythritol powder, (b) biomimetic calcium phosphate (BioCaP) powder, (c) hydroxylapatite (HA) powder, (d) BioCaP–erythritol mixture, (e) HA–erythritol mixture, (f) stabilized EMS AIR‐FLOW tip, and (g) test groups and used powders

We used three different types of powders, two of which were a mixture (Figure 2):

1. HA–erythritol mixture: HA powder with a mean particle size of about 5 μm was mixed with the erythritol powder with mean particle size about 14 μm. The mixture was made in the ratio of 4% of HA and 96% of erythritol according to their weight. HA powder particles are made up of nanoparticles but form microparticles.
2. BioCaP–erythritol mixture: BioCaP powder is a biomimetic CaP powder with a particle size between 15 and 75 μm. This powder is produced under physiological conditions and developed at Academic Centre for Dentistry Amsterdam (Liu et al., 2013). This powder is mixed with erythritol in the ratio of 4% of BioCaP and 96% of erythritol.
3. Erythritol (pure) powder: Erythrithol powder is used as the commercially available form (EMS AIR‐FLOW Powder Plus).

The treatment consisted of two subsequent steps. The goal of Step 1 was cleaning. The goal of Step 2 was modification of the surface by BioCaP coating. The pressure inside the powder chamber was 240,000 Pa (2.4 bar) for all steps. The water flow changed for each step: HA (mixture) cleaning: 42 ml/min, BioCaP (mixture) Coating 1: 2 ml/min, and BioCaP (mixture) Coating 2: 15 ml/min. The test groups were as follows:

Group 1: HA (mixture) cleaning + BioCaP (mixture) Coating 1; Group 2: HA (mixture) cleaning + BioCaP (mixture) Coating 2; Group 3: erythritol (pure) cleaning + BioCaP (mixture) Coating 1; Group 4: erythritol (pure) cleaning + BioCaP (mixture) Coating 2; Group 5: HA (mixture) cleaning + no coating; Group 6: erythritol (pure) cleaning; and control group: cleaning without powder.

During the application, the airflow nozzle was fixed with an angle of 60° and a distance of 4 mm to the disc. The nozzle was not moved during the application, but the disc was moved circularly parallel to the ground to let the air spray uniformly and reach the whole surface. The duration of the cleaning step was open ended. The cleaning step continued until the application was ineffective, meaning either there was no visible biofilm left or the remaining biofilm was not detaching any further despite prolonged application. Therefore, the application time varied in each disc, but the approximate time needed was 1 min. The coating step was applied for 30 s.

### 2.3 Initial and residual biofilm measurement

The cleaning effect was measured quantitatively on titanium discs. All the disc surfaces were 100% covered by biofilm. The biofilm was made visible using erythrosine dye (Figure 1). The biofilm before and after treatment was displayed using a digital camera (Olympus, DF, Plapo, 1× PF, Japan) placed on a stereomicroscope (Olympus SZX 12, Japan). The images were recorded on a color print, and measurement of the percentages of the residual biofilm area was performed using the point‐counting measurement methodology described by Cruz‐Orive and Weibel (1990). All measurements were performed by a blinded examiner.

### 2.4 Surface structure and chemical content

The surface structure of the discs and implants were examined using SEM (XL20, Fei Company, Eindhoven, The Netherlands). The SEM pictures were taken before and after the treatment. The chemical composition of the surface of the specimens were analyzed using energy‐dispersive X‐ray spectroscopy (EDS, EDAX, phoenix system, Tilburg, The Netherlands). Furthermore, implants lost due to peri‐implantitis were analyzed by SEM and EDS before and after treatment. The surface structures and biofilm were visualized.

### 2.5 Statistical analyses

In order to compare the cleaning effect between groups, a pairwise Student's *t* test was used in which we assessed whether the means of two groups were statistically different from each other. The significance level was fixed at *p* < .05.

## 3 RESULTS

### 3.1 Cleaning effect

**Figure 3:**
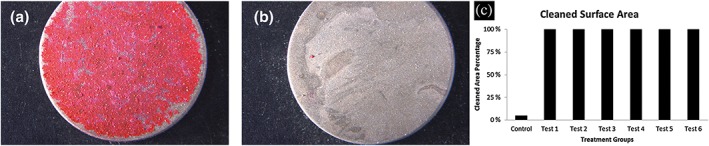
(a and b) Posttreatment light microscope photo of a titanium discs. (a) Control group, (b) Test Group 1, and (c) cleaned surface area percentage per group in column graph

The mean of the cleaned surface area percentage for all test groups was 100%, whereas it was 5% for the control group (Figure 3a,b). Both powders were equally effective and showed statistically significantly better cleaning compared to the control group without powder (Figure 3c). The average time needed to clean one disc was 21 s and did not show differences between the two powders. The average consumed powder amount was 0.41 g for the HA powder and 0.54 g for the erythritol powder.

### 3.2 Pretreatment and posttreatment surface structure of titanium discs

The pretreatment surface of the titanium discs was covered with clusters of Ca(OH)_2_ precipitation as shown in Figure 5f. The layer concealed the SLA surface structure. The EDS analyses showed that the chemical content contained Ca, Mg, S, Si, and P, which was similar to explanted implants.

The posttreatment surface of the treated discs was free of biofilm. The surface structures were clearly visible. The characteristics of the SLA surface such as the grooves and sharp edges were unchanged.

**Figure 4:**
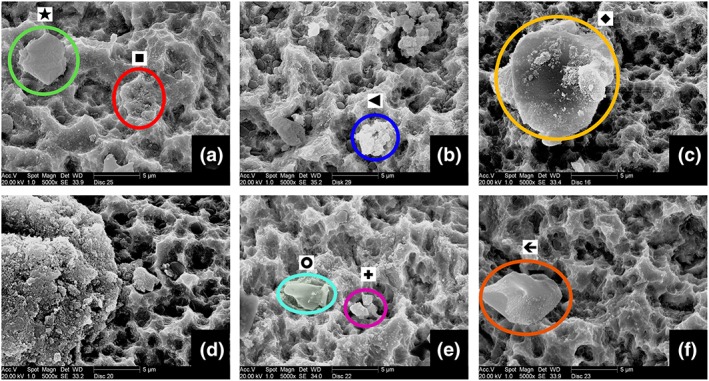
Scanning electron microscopy photos of cleaned titanium discs. (a) Group 1—green circle (star): BioCaP particle red circle (square): HA particles, (b) Group 2—blue circle (triangle): HA particles, (c) Group 3—yellow circle (rhombus): BioCaP particle, (d) Group 4—left‐hand side: BioCaP powder particle residue, (e) Group 5—turquoise circle (circle): erythritol powder particle pink circle (plus): HA particle, (e) Group 6—orange circle (arrow): erythritol powder particle

All discs, independent of the treatment, showed powder particles embedded on the titanium surface. Their shape and size varied depending on the kind of the powder used. The amount and the distribution of particles showed differences among groups (Figure 4).

The groups with HA cleaning (Groups 1, 2, and 5) showed many small sized (less than 1 μm), round particles spread on the surface. These particles were attached to the titanium surface, including the deep grooves. The HA powder particles are microparticles that are made up of nanoparticles. When they hit the surface, they split in pieces and results in this view. Looking at the size and the shape, these particles were expected to be HA nanoparticles. The EDS analyses, performed afterwards, confirmed this expectation by showing the chemical content.

The HA cleaning groups that had an additional coating step showed bigger size CaP particles left on the surface besides the nanoparticles. The shape and size of these bigger particles morphologically resembled the BioCaP powder. Apart from the morphological similarity, the chemical content was shown to be Ca and P by EDS analyses, confirming that they were BioCaP powder particles.

The groups with erythritol cleaning (Groups 3, 4, and 6) showed a different pattern of powder spread on the surface. The discs in these groups did not show the small‐sized particles embedded on the surface. Instead, bigger and square‐like particles were attached on the surface. Among these three groups, the ones that had an additional coating step (Groups 3 and 4) showed a reasonable amount of round and smaller particles, which were different than the group without the coating (Group 6). The size of these round particles ranged between 1 and 10 μm, whereas the square‐like particles were around 20 μm. According to the EDS analyses, the round particles were CaP. However, the square‐like particles could not be detected by EDS because EDS cannot detect organic content. This was a sign confirming that these particles were erythritol powder particles.

All disc surfaces except Group 6 showed Ca and P according to the general chemical content analyses performed by EDS.

### 3.3 Pretreatment and posttreatment implant surface

The in situ peri‐implantitis biofilm on the explanted implant showed the rod and cocci bacteria inside a slime structure on SEM photos. The biofilm mass was so thick that the SLA titanium surface could not be observed.

**Figure 5:**
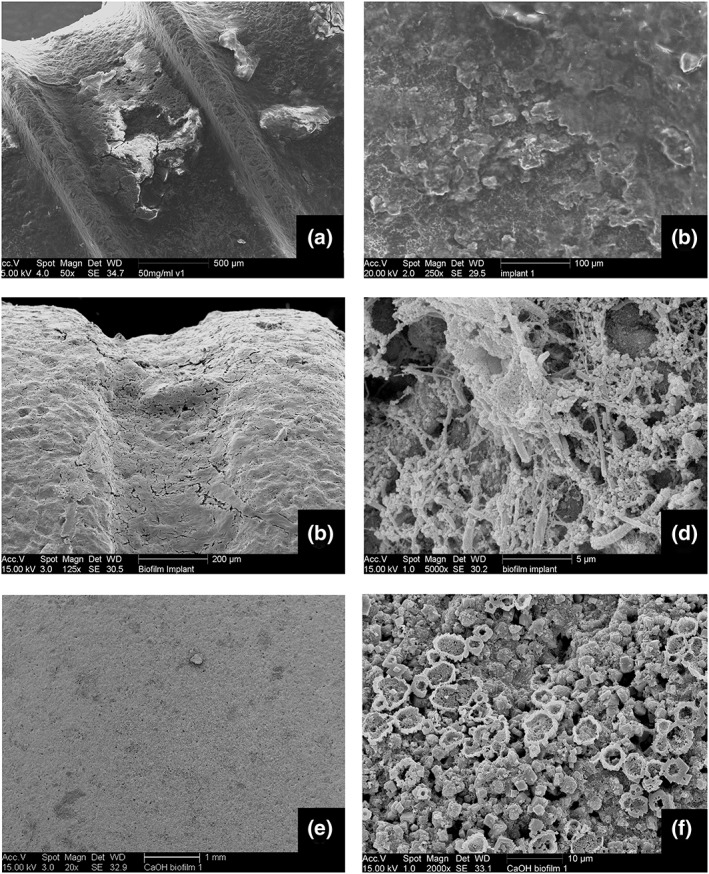
(a and b) Explanted implant surface mineral accumulations. (c and d) Freshly fixed biofilm on a peri‐implantitis exposed biofilm. (e and f) CaOH accumulated in vitro biofilm on a titanium disc

The pretreatment surface of explanted implants showed irregularities and mineral accumulations (Figure 5). The chemical content of the surface consisted of Ca, S, Si, and P, whereas the mineral accumulations consisted of mainly Ca and P.

**Figure 6:**
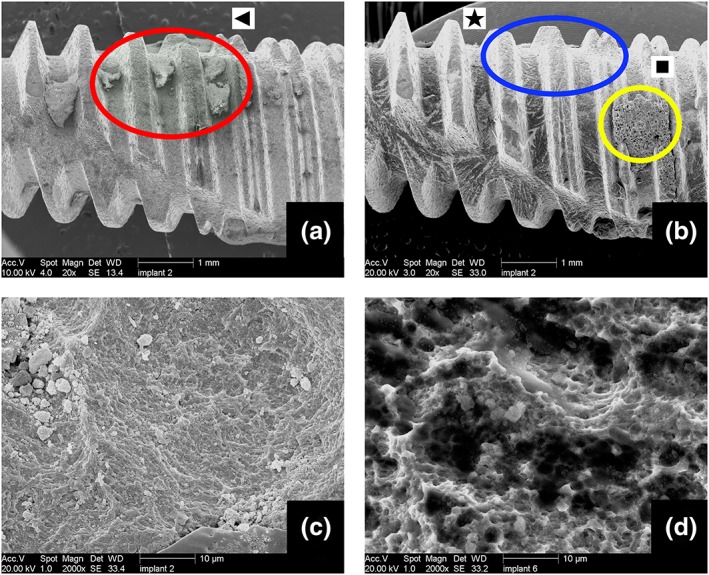
(a) Pretreatment photo of the implant showing calculus on the threads (red circle [triangle]). (b) Posttreatment photo of the same implant after “erythritol cleaning + low coating.” Large calculus is removed (blue circle [star]); however, some powder accumulations are visible on the treated implant surface (yellow circle [square]). (c) Large magnification Scanning electron microscopy photo showing the powder particles embedded on the surface of the same implant. (d) Large magnification scanning electron microscopy photo of another implant showing the biofilm remnant and powder particles on the surface

The posttreatment SEM photos showed that subsequent to the cleaning treatment step, most of the large calculus on the implants was removed (Figure 6). However, the large and thick calculus was only superficially removed, leaving a thin layer attached on the surface of some of the implants. No difference was observed between HA and erythritol powder cleaning regarding this aspect.

The surface of some of the implants showed clusters of powder particles that were attached and covered the surface. This type of accumulation was observed on the discs that were treated with less water flow. This means that less water flow correspond to more powder being accumulated on the surface.

With larger magnification SEM photos, the CaP powder particles were observed on the titanium surface. The SLA structure was not damaged and was mostly free of biofilm.

## 4 DISCUSSION

This study showed that APA treatment with biocompatible powders under intraorally applicable pressure settings efficiently cleaned Ca‐precipitated organic film‐coated titanium discs and explanted implants. The surface morphology remained mostly unchanged with exceptions of powder particles embedded on titanium.

The applied treatment consisted of two steps each with different goals. Step 1 aimed to clean the biofilm, and Step 2 aimed to modify the surface to improve the osteoconductive properties of the surface. The reasons of separating these aims into two steps are that firstly, we use different powders that are suitable for each purpose, and secondly, the surface modification should be done on a biofilm‐free surface; otherwise, the remaining biofilm might impair the osteoconductive potential.

The first aim of this study was to discover if biocompatible powders would efficiently clean the surface under intraorally applicable pressure settings. The pressure and water settings used here are suitable for subgingival application. There are many in vivo studies showing that the subgingival application of the same APA device under these settings is safe (Ji et al., 2014; Sahm, Becker, Santel, & Schwarz, 2011; Schou et al., 2003). Two different water settings were applied for coating steps. Because high water flow was rinsing away the excessive powder, the amount of powder particles attaching on the surface was expected to be dependent on the water settings. Therefore, the coating step was applied with different settings; however, the surface modification did not show significant differences.

There were two powder modifications in this study. The coating step powder is a mixture of BioCaP and erythritol powder. BioCaP is a biocompatible and degradable CaP produced by precipitation in simulated body fluids under physiological conditions (37 °C; Liu et al., 2013). The in vitro and in vivo studies reported good physiochemical and biocompatible properties and the in vivo degradation of the material (Liu et al., 2013). Because of the high biocompatibility of the material, no adverse effects in the body were expected. The usage of the proportion of 4% BioCaP and 96% erythritol was determined on the basis of the physical properties of the powders. The soft nature of an unsintered BioCaP powder was only poorly compatible with the EMS device. By using pure BioCaP powder, we experienced many nozzle‐clogging issues, whereas the mixture with 4% BioCaP and 96% erythritol did not show this problem. Another reason for the usage of 4% BioCaP proportion instead of 100% BioCaP was the aim to produce a titanium surface with rare CaP particles embedded. This was based on the results of a pilot study we performed, which showed us the following: The cell response towards a thick visible CaP layer was not as good as a surface that has CaP particles embedded on the SLA titanium surface. With the help of the mixture, the excessive loading of the CaP powder on the surface was prevented, and surface modification was achieved as confirmed by the SEM pictures (Figure 6). However, whether the attached particles are able to create the intended osteoconductive properties needs to be shown by further in vitro cell response experiments and even by animal studies.

The other powder mixture included HA powder. HA is a nondegradable powder that contains hard particles. The hard powders are expected to cause harm when sprayed on a surface; however, according to a previous study, the damaging effect depends on the particle size (Tada, Kakuta, Ogura, & Sato, 2010). The small‐sized particles do not cause a surface damage when sprayed on a surface. For this reason, we used a particle size of 5 μm for HA application. In addition to that, the amount of HA in the mixture was kept at 4%. This minimized the damaging effect of the hard powder. Furthermore, a number of animal studies on surgical APA application with sodium bicarbonate—that is another hard powder—did not report complications (Schou et al., 2003; Deppe, Horch, Henke, & Donath, 2001). The supposed safety of this treatment should be tested with further in vivo studies.

Instead of a fixed application time, an open‐ended duration of cleaning was performed. The cleaning was performed until it was ineffective. The reason for choosing this methodology was to show the time‐independent effect of the treatment. As shown in the control group, if the treatment itself was ineffective, the biofilm would not be removed no matter how long it was applied, which is probably due to the fact that the film layer on the discs was a very strongly attaching layer. On the other hand, if a weak biofilm model was used, by applying the cleaning too long, the cleaning effect of all the treatments would be equalized. However, this was not the case in our model. Therefore, we did not limit the application time and observed how long it took to clean the disc with each treatment.

The second aim of this study was to create a modified titanium surface that is attractive to bone tissue. After the treatment, the titanium was covered with osteoconductive powder particles spread and impacted on the surface. We speculate that these HA and CaP particles will stimulate bone growth towards the implant surface. Being similar to the bone mineral in composition, CaPs are bioactive and able to achieve an early and functional bone apposition on the implants (Chow, 2001; Chow & Eanes, 2001; Driessens, 1982; Kd, 1983; LeGeros, 1991; LeGeros, 2002). The release of calcium and phosphate ions into the peri‐implant region increases the saturation of body fluids, leading to the precipitation of a biological carbonated apatite onto the surface of the implant (Ducheyne, Radin, & King, 1993; Ducheyne & Qiu, 1999; Radin & Ducheyne, 1993) This layer of biological apatite might incorporate endogenous proteins and serve as a matrix for osteogenic cells' attachment and growth. These cells produce the bone extracellular matrix, resulting in a direct apposition of bone tissue on the surface. The bone‐healing process around the implant is therefore enhanced by this biological apatite layer (Ducheyne et al., 1993; Ducheyne & Qiu, 1999). Furthermore, studies conducted both in vitro and in vivo have shown that BCP grit‐blasted surfaces promoted an early osteoblast differentiation, bone apposition, and greater bone‐to‐implant contact as compared to smooth or alumina grit‐blasted titanium implants (Citeau, Guicheux, Vinatier, et al., 2005; Le Guehennec et al., 2008). However, one of the major concerns with certain CaP coatings is the possible delamination of the coating from the surface of the titanium implant and failure at the implant/coating interface. These negative publications have resulted in the development of thin CaP‐coating techniques that can overcome this problem. Depending on this knowledge, we speculate that our model has the advantage of the initial positive effect of CaP particles but do not have the risk of having the long‐term problems. This is because the BioCaP is a fast biodegradable CaP (Liu et al., 2013) and we do not create a thick coating layer on the surface, which has the risk of delamination. However, further studies showing the response of osteoblast‐like cells towards the surface are needed to prove this theory.

As it is very difficult to mimic the peri‐implantitis biofilm in vitro, one limitation of the study was to have a realistic biofilm model. The model required a very strongly attached in vitro biofilm. The ideal biofilm should not be removed by the air and water spray—when applied without powder—as our aim was to see the effect of the abrasive powder. The intraoral biofilm that we used in our previous study met this requirement because it had this property. However, because of the difficulties of the production of this biofilm, it was not possible to produce a sufficient number of discs for this study. We had to find an in vitro model that allowed us to produce comparable discs. After testing several different in vitro biofilm models, we found that most of these biofilms were removed easily by water and air spray even without using any powder. This made it impossible to test the effect of our powders (Supporting Information). After several modifications, we found that Ca precipitation on an organic film layer met our expectations. Because the Ca precipitation changes the properties of the biofilm, we preferred not to call the model a “biofilm” but a “Ca‐precipated organic film.” This model not only contains the Ca content that is present in calculus on the surface of the infection‐exposed implants but also includes the organic layer with glycoproteins and bacteria. To achieve this aim, firstly, the discs were preincubated with 20% FBS‐containing medium. According to a study by Lima, Koo, Vacca Smith, Rosalen, and Del Bel Cury (2008), it was shown that more bacteria adhere to serum‐coated titanium than uncoated titanium. This pellicle formation facilitated the biofilm formation. Unstimulated saliva is used as an inoculum instead of a single‐species bacteria inoculum because peri‐implantitis is not an infection caused by single pathogens; rather, it is a multimicrobial infection (Koban, Holtfreter, Hubner, et al., 2011). Furthermore, *Streptococcus mutans* monospecies biofilm was one of the models tried in our pilot experiments. This biofilm was removed easily by even gentle wiping (Supporting Information). Therefore, we showed that it was not suitable for our study model, which is in line with a former study. Koban et al. (2011) showed that *S. mutans* monospecies biofilms do not present a suitable model for biofilms on dental implants. The medium was refreshed every 3 days, which is less frequent than the common biofilm models. This was done to avoid the formation of a thick and, thus, loose biofilm. According to the literature, frequent medium refreshment results in fast‐growing bacteria and slime layer and therefore a very thick and easily detachable biofilm (Donlan, 2002). However, our priority was to have a firm organic layer rather than a thick one.

The peri‐implantitis exposed implant surface shows high mineral content, specifically calcium (Esposito, Lausmaa, Hirsch, & Thomsen, 1999; Shibli, Vitussi, Garcia, et al., 2007). The mineralization of the biofilm is one of the reasons for the difficulty of cleaning the implant surface. The additional Ca precipitation on the discs in our model aims to imitate this aspect based on the role of calcium in biofilm formation and activity reported in the article by van der Waal and van der Sluis (2012). It is hypothesized that calcium plays an important role in scaffolding an endodontic biofilm and consequently will be incorporated in the structure of its extracellular matrix (van der Waal & van der Sluis, 2012).

After our in vitro test, we aimed to test the cleaning in a more clinically relevant model. Therefore, we employed actual peri‐implantitis‐exposed implants. This model was closer to the real clinical situation from two aspects. First of all, the deposits on the surface were the real intraoral calculus. Secondly, real implants were used as specimens. This means that the cleaning was tested on a more challenging macrostructure compared to the discs.

The two models used in this study, namely, the in vitro and ex vivo models, showed certain similarities and differences to each other and to the in vivo peri‐implantitis biofilm. In the second part of the study, dried explanted implants were used. These implants had large calculus on the surface of which content was mostly Ca, resembling the Ca‐precipitated organic film on the discs. However, the in vitro model used in the first part of the study has clusters of Ca on the surface, whereas the implants do not have. Although the content was similar, the morphology was different. On the other hand, the fact that the implants were autoclaved and dried prior to the treatment was an obvious shortcoming of the model. However, the implants did not undergo a mechanical cleaning before sterilization. Therefore, the autoclaving did not damage the calculus remnants on the surface. The air drying following the sterilization makes the biofilm firmer attached to the surface due to the loss of water. As a finding of our pilot experiments, we observed that it is more difficult to remove a dried biofilm than a fresh one. Hence, although this was a shortcoming of the model, it actually made the task more challenging for our treatment. In spite of this fact, our cleaning treatment was able to remove most of the calculus including the ones on the deep grooves that were difficult to reach. Therefore, we speculate that the treatment could be more efficient during a possible in vivo application, considering that the fresh peri‐implatitis biofilm on site is wet and softer than our specimens. On the other hand, the surgical site has anatomical limitations that make it difficult to reach certain parts of the implant surface. These factors also play a role on the in vivo success of the treatment.

## 5 CONCLUSIONS

APA treatment with osteoconductive CaP and HA powders under safe (intraorally applicable) pressure settings is efficient in cleaning in vitro Ca‐precipitated biofilms on the titanium SLA surface discs and explanted implants. The treatment results in a surface modification showing impacted powder particles.

## FUNDING

The work was supported by the Department of Oral Implantology, Academical Center of Dentistry Amsterdam, University of Amsterdam and Vrije Universiteit Amsterdam University, The Netherlands.

## CONFLICT OF INTEREST

Ceylin S. Tastepe declares that he has no conflict of interest. Xingnan Lin declares that he has no conflict of interest. Arie Werner declares that he has no conflict of interest. Marcel Donnet is an engineer working in the research group of E.M.S. company. He has acted as a scientific consultant for the technical and chemical issues regarding the devices and the nature of the powders used. He had no influence on the data collection nor the publishing. Daniel Wismeijer declares that he has no conflict of interest. Yuelian Liu declares that he has no conflict of interest.

## ETHICAL APPROVAL

This article does not contain any studies with human participants or animals performed by any of the authors.

## INFORMED CONSENT

For this type of study, formal consent is not required.

## Supporting information

Table 1. Biofilm models
Table 2. The responds of the different biofilm models to different cleaning methods. The results of the biofilm removal per treatment is described by signs. (+++): Not removed, (++): Not Removed Easily, (−): Removed, (−‐): Removed Relatively Easily, (−‐‐): Removed Easily.
