# Supplementary material for: Cleaning effect of osteoconductive powder abrasive treatment on explanted human implants and biofilm‐coated titanium discs
Source: Clin Exp Dent Res. 2018 Feb 15;4(1):25–34. doi: 10.1002/cre2.100 (PMC5813889; doi:10.1002/cre2.100)
Supplement: Supplementary file 1 — Table 1. Biofilm models Table 2. The responds of the different biofilm models to different cleaning methods. The results of the biofilm removal per treatment is described by signs. (+++): Not removed, (++): Not Removed Easily, (−): Removed, (−‐): Removed Relatively Easily, (−‐‐): Removed Easily. [file CRE2-4-25-s001.docx]

**Pilot Biofilm Experiments**

**Background**

The peri-implantitis exposed implants are covered with a strongly attaching biofilm which is not easily removed by different treatment methods [2]. However the in vitro biofilm models are not strong enough to test the mechanical surface cleaning methods.

Aim: The aim of this pilot experiment was to develop a strongly attaching biofilm model which cannot be detached easily by mechanical methods and should be mimicking the mineral content of the peri-implantitis biofilm.

**Materials and Methods**

Nine different biofilm models are established using different inoculum , medium type and time, either with or without calcification. The details of the models can be seen on table 1. The biofilms were created on titanium discs and stained and mechanically cleaned subsequently. They were examined under light microscope before and after cleaning.

**Table 1. Biofilm models**

| **Model No** | **Inoculum** | **Medium** | **Time** | **Calcification (**Ca(OH)_2_ **)** |
| --- | --- | --- | --- | --- |
| 1 | S. Mutans (C180-2) | 0.2% sucrose+ McBain Medium | 48 hour |  |
| 2 | Saliva | No sucrose + (α-MEM) Medium | 48 hour | - |
| 3 | Saliva | 0.2% sucrose+ McBain Medium | 7 days | 48 hours |
| 4 | Saliva | 0.5% sucrose+ McBain medium | 7 days | 48 hours |
| 5 | Saliva + S. Mutans (C180-2) | 0.2% sucrose+ McBain Medium | 7 days | 48 hours |
| 6 | Saliva + S. Mutans (C180-2) | 0.5% sucrose+ McBain medium | 7 days | 48 hours |
| 7 | S. Mutans (C180-2) | 0.2% sucrose+ McBain Medium | 7 days | 48 hours |
| 8 | S. Mutans (C180-2) | 0.5% sucrose+ McBain medium | 7 days | 48 hours |
| 9 | Saliva | No sucrose + (α-MEM) Medium | 7 days | 48 hours |

**Results**

The attachment strength of the biofilms was tested by removing the biofilm with different mechanical cleaning methods: 1- Simple water spray 2- Cotton pellet wiping 3- EMS Air Flow Device Perio Flow Mode 9 LED pressure- Highest Water without powder 4- EMS Air Flow Device Perio Flow Mode 9 LED pressure- Highest Water with powder (Table 2). Biofilm model 1 and 2 were so weak that they could be removed with any of the above mentioned methods including the most gentle rinsing. This showed us that it is impossible to test the cleaning efficiency of our method with the model 1 and 2. Model 9 and 4 were the strongest biofilms however model 4 could be cleaned in a shorter time than model 9. We believe that it is because model 4 was a thicker biofilm which has a bigger tendency to detach from the surface easily [1]. Therefore, we selected Model 9 for our experiments. This model is described below.

**Table 2. The responds of the different biofilm models to different cleaning methods. The results of the biofilm removal per treatment is described by signs. (+++): Not removed, (++): Not Removed Easily,**

**(-): Removed, (--): Removed Relatively Easily, (---): Removed Easily .**

| Model No | Simple water Spray | Cotton Pellet wiping | Air Flow 9LED pressure Highest Water – No Powder | Air Flow 9LED pressure Highest Water – EMS Powder |
| --- | --- | --- | --- | --- |
| 1 | **-** | **---** | **---** | **---** |
| 2 | **-** | **---** | **---** | **---** |
| 3 | **+++** | **-** | **++** | **-** |
| 4 | **+++** | **++** | **+++** | **-** |
| 5 | **+++** | **--** | **--** | **-** |
| 6 | **+++** | **--** | **--** | **-** |
| 7 | **+++** | **---** | **-** | **-** |
| 8 | **+++** | **---** | **-** | **-** |
| 9 | **+++** | **++** | **+++** | **-** |

**References**

1. Donlan RM (2002) Biofilms: microbial life on surfaces. Emerging infectious diseases 8:881-890

2. Heuer W, Elter C, Demling A et al. (2007) Analysis of early biofilm formation on oral implants in man. J Oral Rehabil 34:377-382
